# *N*-{4-[(*E*)-(4-Methyl­phen­yl)imino­meth­yl]phen­yl}acetamide

**DOI:** 10.1107/S1600536811001887

**Published:** 2011-01-22

**Authors:** M. Nawaz Tahir, Hazoor Ahmad Shad

**Affiliations:** a Department of Physics, University of Sargodha, Sargodha, Pakistan; b Department of Chemistry, Govt. M. D. College, Toba Tek Singh, Punjab, Pakistan

## Abstract

There are two symmetry-independent mol­ecules in the asymmetric unit of the title compound, C_16_H_16_N_2_O, that differ in conformation. The dihedral angles between the benzene rings in the two mol­ecules are 44.35 (19) and 48.14 (17)°, but the rings twist in opposite directions. The acetamide groups make nearly equal dihedral angles of 25.4 (3) and 25.7 (3)° with the parent benzene rings. An *S*(6) ring motif is formed in each mol­ecule by intra­molecular C—H⋯O close contacts. In the crystal, strong N—H⋯O hydrogen bonds between acetamide groups generate a *C*(4) chain motif arranging the mol­ecules into two symmetry-independent polymeric structures extending along [010].

## Related literature

For related structures, see: Karlsen *et al.* (1988 ▶); Tahir *et al.* (2010 ▶). For graph-set notation, see: Bernstein *et al.* (1995 ▶).
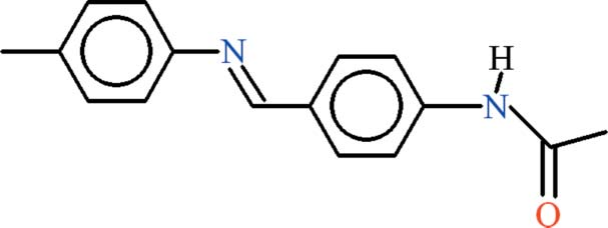


## Experimental

### 

#### Crystal data

C_16_H_16_N_2_O
*M* *_r_* = 252.31
Triclinic, 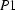

*a* = 7.1044 (8) Å
*b* = 9.7393 (10) Å
*c* = 10.9236 (12) Å
α = 109.731 (5)°
β = 91.799 (6)°
γ = 100.679 (6)°
*V* = 695.51 (13) Å^3^
*Z* = 2
Mo *K*α radiation
μ = 0.08 mm^−1^
*T* = 296 K
0.26 × 0.16 × 0.12 mm

#### Data collection

Bruker Kappa APEXII CCD diffractometer
Absorption correction: multi-scan (*SADABS*; Bruker, 2005 ▶) *T*_min_ = 0.980, *T*_max_ = 0.990
10024 measured reflections
2453 independent reflections
1460 reflections with *I* > 2σ(*I*)
*R*_int_ = 0.059

#### Refinement

*R*[*F* ^2^ > 2σ(*F* ^2^)] = 0.044
*wR*(*F* ^2^) = 0.112
*S* = 0.97
2453 reflections
339 parameters
3 restraints
H-atom parameters constrained
Δρ_max_ = 0.16 e Å^−3^
Δρ_min_ = −0.15 e Å^−3^

### 

Data collection: *APEX2* (Bruker, 2009 ▶); cell refinement: *SAINT* (Bruker, 2009 ▶); data reduction: *SAINT*; program(s) used to solve structure: *SHELXS97* (Sheldrick, 2008 ▶); program(s) used to refine structure: *SHELXL97* (Sheldrick, 2008 ▶); molecular graphics: *ORTEP-3 for Windows* (Farrugia, 1997 ▶) and *PLATON* (Spek, 2009 ▶); software used to prepare material for publication: *WinGX* (Farrugia, 1999 ▶) and *PLATON*.

## Supplementary Material

Crystal structure: contains datablocks global, I. DOI: 10.1107/S1600536811001887/gk2340sup1.cif

Structure factors: contains datablocks I. DOI: 10.1107/S1600536811001887/gk2340Isup2.hkl

Additional supplementary materials:  crystallographic information; 3D view; checkCIF report

## Figures and Tables

**Table 1:** Hydrogen-bond geometry (Å, °)

*D*—H⋯*A*	*D*—H	H⋯*A*	*D*⋯*A*	*D*—H⋯*A*
N2—H2*A*⋯O2^i^	0.86	2.00	2.854 (5)	172
N4—H4⋯O1^ii^	0.86	2.06	2.911 (4)	173
C13—H13⋯O1	0.93	2.40	2.922 (6)	116
C29—H29⋯O2	0.93	2.35	2.864 (6)	114

Symmetry codes: (i) 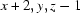; (ii) 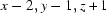.

**Fig. 1.**
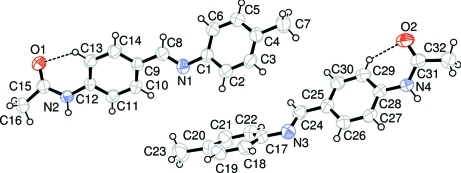
Molecular structure of the title compound with the atom numbering scheme. The displacement ellipsoids are drawn at the 50% probability level. H atoms are shown by small circles of arbitrary radii. The dotted line represent the intramolecular hydrogen bond.

**Fig. 2.**
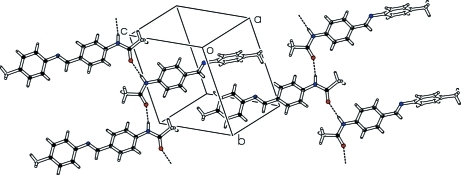
The crystal packing (PLATON; Spek, 2009) showing two symmetry independent one dimensional polymeric chains along the b-axis.

## supplementary crystallographic information

### Comment

The title compound (I, Fig. 1) is being reported as a part of our ongoing project related to the synthesis of various Schiff bases of 4-methylaniline. In this regard recently we have reported the synthesis and crystal structure of *N*-[(*E*)-1,3-benzodioxol-5-ylmethylidene]-4-methylaniline (Tahir *et al.*, 2010).

The crystal structure of thiacetazone *i.e.*, *N*-{4-[-(2-carbamothioylhydrazinylidene)methyl]phenyl}acetamide (Karlsen *et al.*, 1988) has been published which contains the common moiety of *N*-(4-formylphenyl)acetamide as in (I).

The title compound consists of two molecules in the crystallographic asymmetric unit which differ from each other geometrically. In one molecule, the 4-methylaniline group A (C1—C7/N1), groups B (C8—C14) and C (N2/C15/C16/O1) of *N*-(4-formylphenyl)acetamide are planar with r. m. s deviation of 0.0062, 0.0169 and 0.0005 Å, respectively. The dihedral angle between A/B, A/C and B/C is 48.11 (15)°, 25.13 (25)° and 25.88 (27)°, respectively. In second molecule, the 4-methylaniline group D (C17—C23/N3), groups E (C24—C30) and F (N4/C31/C32/O2) of *N*-(4-formylphenyl)acetamide are planar with r. m. s deviation of 0.0292, 0.0146 and 0.0027 Å, respectively. The dihedral angle between D/E, D/F and E/F is 44.36 (17)°, 69.79 (17)° and 25.43 (23)°, respectively. In each molecule there exist S(6) ring motif (Bernstein *et al.*, 1995) due to intramolecular H-bonding of C—H···O type (Table 1, Fig. 1). The molecules are stabilized in the form of one dimensional polymeric chains extending along the crystallographic *b*-axis due to intermolecular hydrogen bonds of N—H···O type (Table 1, Fig. 2). These C(4) chains  (Bernstein *et al.*, 1995) are formed via interaction of the amide groups. There does not exist any kind of strong π-interaction.

### Experimental

Equimolar quantities of 4-methylaniline and *N*-(4-formylphenyl)acetamide were refluxed in methanol along with a  few drops of acetic acid as catalyst for 30 min resulting in colorless solution. The solution was kept at room temperature. After six days colourless needles of the title compound were isolated.

### Refinement

In the absence of anomalous scattering all Friedal pairs were merged. All H atoms were positioned geometrically (N–H = 0.86, C–H = 0.93–0.96 Å) and were included in the refinement in the riding model approximation, with *U*_iso_(H) = *xU*_eq_(C, N), where *x* = 1.5 for methyl H-atoms and *x* = 1.2 for all other H-atoms.

### Crystal data

**Table:**

C_16_H_16_N_2_O	*Z* = 2
*M**_r_* = 252.31	*F*(000) = 268
Triclinic, *P*1	*D*_x_ = 1.205 Mg m^−^^3^
Hall symbol: P 1	Mo *K*α radiation, λ = 0.71073 Å
*a* = 7.1044 (8) Å	Cell parameters from 1460 reflections
*b* = 9.7393 (10) Å	θ = 2.0–25.1°
*c* = 10.9236 (12) Å	µ = 0.08 mm^−^^1^
α = 109.731 (5)°	*T* = 296 K
β = 91.799 (6)°	Needle, colorless
γ = 100.679 (6)°	0.26 × 0.16 × 0.12 mm
*V* = 695.51 (13) Å^3^	

### Data collection

**Table:**

Bruker Kappa APEXII CCD diffractometer	2453 independent reflections
Radiation source: fine-focus sealed tube	1460 reflections with *I* > 2σ(*I*)
graphite	*R*_int_ = 0.059
Detector resolution: 8.20 pixels mm^-1^	θ_max_ = 25.1°, θ_min_ = 2.0°
ω scans	*h* = −8→8
Absorption correction: multi-scan (*SADABS*; Bruker, 2005)	*k* = −10→11
*T*_min_ = 0.980, *T*_max_ = 0.990	*l* = −13→13
10024 measured reflections	

### Refinement

**Table:**

Refinement on *F*^2^	Primary atom site location: structure-invariant direct methods
Least-squares matrix: full	Secondary atom site location: difference Fourier map
*R*[*F*^2^ > 2σ(*F*^2^)] = 0.044	Hydrogen site location: inferred from neighbouring sites
*wR*(*F*^2^) = 0.112	H-atom parameters constrained
*S* = 0.97	*w* = 1/[σ^2^(*F*_o_^2^) + (0.052*P*)^2^] where *P* = (*F*_o_^2^ + 2*F*_c_^2^)/3
2453 reflections	(Δ/σ)_max_ < 0.001
339 parameters	Δρ_max_ = 0.16 e Å^−^^3^
3 restraints	Δρ_min_ = −0.15 e Å^−^^3^

### Special details

**Table:**

Geometry. Bond distances, angles *etc*. have been calculated using the rounded fractional coordinates. All su's are estimated from the variances of the (full) variance-covariance matrix. The cell e.s.d.'s are taken into account in the estimation of distances, angles and torsion angles
Refinement. Refinement of *F*^2^ against ALL reflections. The weighted *R*-factor *wR* and goodness of fit *S* are based on *F*^2^, conventional *R*-factors *R* are based on *F*, with *F* set to zero for negative *F*^2^. The threshold expression of *F*^2^ > σ(*F*^2^) is used only for calculating *R*-factors(gt) *etc*. and is not relevant to the choice of reflections for refinement. *R*-factors based on *F*^2^ are statistically about twice as large as those based on *F*, and *R*- factors based on ALL data will be even larger.

### Fractional atomic coordinates and isotropic or equivalent isotropic displacement parameters (Å^2^)

**Table:**

	*x*	*y*	*z*	*U*_iso_*/*U*_eq_	
O1	1.4696 (5)	1.0625 (3)	−0.3349 (3)	0.0708 (16)	
N1	0.7543 (5)	0.8296 (4)	0.1036 (4)	0.0574 (17)	
N2	1.4290 (5)	0.8628 (4)	−0.2684 (4)	0.0507 (14)	
C1	0.5901 (7)	0.8407 (5)	0.1748 (5)	0.0533 (17)	
C2	0.4818 (7)	0.7111 (5)	0.1826 (5)	0.0581 (19)	
C3	0.3211 (7)	0.7168 (5)	0.2509 (5)	0.0609 (19)	
C4	0.2630 (7)	0.8479 (5)	0.3122 (5)	0.057 (2)	
C5	0.3734 (7)	0.9758 (5)	0.3047 (5)	0.066 (2)	
C6	0.5329 (7)	0.9738 (5)	0.2353 (5)	0.063 (2)	
C7	0.0872 (8)	0.8530 (7)	0.3878 (6)	0.082 (3)	
C8	0.7950 (7)	0.9097 (5)	0.0340 (5)	0.0546 (17)	
C9	0.9643 (7)	0.9052 (5)	−0.0396 (4)	0.0488 (17)	
C10	1.1009 (7)	0.8263 (5)	−0.0238 (5)	0.0560 (17)	
C11	1.2543 (7)	0.8186 (5)	−0.0978 (4)	0.0527 (17)	
C12	1.2758 (6)	0.8876 (5)	−0.1894 (4)	0.0461 (17)	
C13	1.1414 (6)	0.9688 (5)	−0.2060 (4)	0.0525 (17)	
C14	0.9869 (7)	0.9764 (5)	−0.1296 (5)	0.0568 (17)	
C15	1.5113 (6)	0.9450 (5)	−0.3384 (4)	0.0530 (17)	
C16	1.6605 (5)	0.8800 (3)	−0.4230 (4)	0.0656 (19)	
O2	−0.4539 (3)	0.5959 (2)	0.7256 (3)	0.0861 (18)	
N3	0.2922 (3)	0.3139 (2)	0.2969 (3)	0.0618 (17)	
N4	−0.3891 (3)	0.3652 (2)	0.6710 (3)	0.0590 (17)	
C17	0.4543 (7)	0.3342 (5)	0.2265 (5)	0.055 (2)	
C18	0.4305 (7)	0.2583 (5)	0.0940 (5)	0.0638 (19)	
C19	0.5805 (8)	0.2744 (5)	0.0169 (5)	0.0649 (19)	
C20	0.7579 (8)	0.3651 (6)	0.0740 (6)	0.061 (2)	
C21	0.7813 (7)	0.4324 (6)	0.2065 (6)	0.073 (2)	
C22	0.6329 (7)	0.4202 (5)	0.2829 (5)	0.0637 (17)	
C23	0.9201 (8)	0.3872 (7)	−0.0095 (7)	0.093 (3)	
C24	0.2638 (7)	0.4188 (5)	0.3945 (5)	0.060 (2)	
C25	0.0999 (7)	0.4058 (5)	0.4693 (5)	0.0534 (17)	
C26	−0.0233 (8)	0.2677 (5)	0.4452 (5)	0.067 (2)	
C27	−0.1822 (8)	0.2570 (5)	0.5138 (5)	0.063 (2)	
C28	−0.2240 (7)	0.3849 (5)	0.6071 (5)	0.0509 (19)	
C29	−0.1015 (7)	0.5213 (5)	0.6306 (5)	0.0594 (19)	
C30	0.0582 (7)	0.5293 (5)	0.5624 (5)	0.0601 (19)	
C31	−0.4956 (7)	0.4670 (5)	0.7260 (5)	0.0609 (19)	
C32	−0.6685 (8)	0.4123 (6)	0.7853 (6)	0.082 (3)	
H2	0.51695	0.62027	0.14207	0.0694*	
H2A	1.47558	0.78614	−0.27236	0.0609*	
H3	0.24979	0.62868	0.25548	0.0730*	
H5	0.33978	1.06663	0.34770	0.0789*	
H6	0.60181	1.06190	0.22925	0.0763*	
H7A	−0.01968	0.85851	0.33489	0.1221*	
H7B	0.11396	0.93911	0.46638	0.1221*	
H7C	0.05609	0.76452	0.40979	0.1221*	
H8	0.71485	0.97357	0.02934	0.0652*	
H10	1.08883	0.77836	0.03701	0.0673*	
H11	1.34542	0.76574	−0.08575	0.0631*	
H13	1.15408	1.01698	−0.26662	0.0630*	
H14	0.89694	1.03103	−0.13976	0.0682*	
H16A	1.61524	0.85273	−0.51349	0.0982*	
H16B	1.68204	0.79327	−0.40626	0.0982*	
H16C	1.77890	0.95295	−0.40297	0.0982*	
H4	−0.42705	0.27822	0.67571	0.0708*	
H18	0.31304	0.19551	0.05503	0.0760*	
H19	0.56128	0.22404	−0.07300	0.0775*	
H21	0.90168	0.48860	0.24676	0.0868*	
H22	0.65334	0.47021	0.37281	0.0761*	
H23A	0.95401	0.49052	−0.00053	0.1393*	
H23B	0.87856	0.32785	−0.09943	0.1393*	
H23C	1.03017	0.35732	0.01878	0.1393*	
H24	0.35200	0.50929	0.42033	0.0709*	
H26	0.00187	0.18264	0.38272	0.0806*	
H27	−0.26223	0.16437	0.49809	0.0755*	
H29	−0.12660	0.60711	0.69199	0.0708*	
H30	0.14046	0.62137	0.57995	0.0723*	
H32A	−0.75807	0.47678	0.79349	0.1221*	
H32B	−0.62896	0.41233	0.87019	0.1221*	
H32C	−0.72896	0.31278	0.73023	0.1221*	

### Atomic displacement parameters (Å^2^)

**Table:**

	*U*^11^	*U*^22^	*U*^33^	*U*^12^	*U*^13^	*U*^23^
O1	0.085 (3)	0.051 (2)	0.086 (3)	0.0129 (18)	0.032 (2)	0.0348 (19)
N1	0.051 (3)	0.062 (3)	0.063 (3)	0.010 (2)	0.015 (2)	0.027 (2)
N2	0.052 (2)	0.044 (2)	0.061 (3)	0.0141 (18)	0.013 (2)	0.022 (2)
C1	0.051 (3)	0.060 (3)	0.057 (3)	0.016 (2)	0.012 (3)	0.028 (3)
C2	0.060 (3)	0.058 (3)	0.064 (4)	0.016 (2)	0.010 (3)	0.029 (3)
C3	0.061 (3)	0.058 (3)	0.075 (4)	0.011 (2)	0.013 (3)	0.038 (3)
C4	0.051 (3)	0.071 (4)	0.057 (4)	0.018 (3)	0.007 (3)	0.030 (3)
C5	0.071 (4)	0.058 (3)	0.072 (4)	0.020 (3)	0.019 (3)	0.022 (3)
C6	0.066 (4)	0.052 (3)	0.073 (4)	0.006 (3)	0.021 (3)	0.025 (3)
C7	0.071 (4)	0.104 (5)	0.083 (4)	0.023 (3)	0.024 (4)	0.046 (4)
C8	0.051 (3)	0.057 (3)	0.056 (3)	0.011 (2)	0.008 (3)	0.020 (3)
C9	0.047 (3)	0.051 (3)	0.051 (3)	0.010 (2)	0.008 (3)	0.021 (2)
C10	0.064 (3)	0.058 (3)	0.053 (3)	0.014 (3)	0.014 (3)	0.027 (3)
C11	0.057 (3)	0.055 (3)	0.052 (3)	0.014 (2)	0.005 (3)	0.025 (3)
C12	0.044 (3)	0.049 (3)	0.046 (3)	0.008 (2)	0.008 (3)	0.018 (2)
C13	0.053 (3)	0.052 (3)	0.057 (3)	0.010 (2)	0.008 (3)	0.025 (2)
C14	0.053 (3)	0.062 (3)	0.062 (3)	0.014 (2)	0.006 (3)	0.029 (3)
C15	0.055 (3)	0.047 (3)	0.050 (3)	0.001 (2)	0.006 (3)	0.013 (2)
C16	0.061 (3)	0.069 (3)	0.073 (4)	0.014 (3)	0.024 (3)	0.031 (3)
O2	0.081 (3)	0.060 (2)	0.133 (4)	0.0285 (18)	0.030 (2)	0.045 (2)
N3	0.066 (3)	0.056 (3)	0.064 (3)	0.011 (2)	0.014 (3)	0.022 (2)
N4	0.064 (3)	0.050 (3)	0.075 (3)	0.021 (2)	0.023 (3)	0.031 (2)
C17	0.061 (4)	0.045 (3)	0.060 (4)	0.010 (2)	0.011 (3)	0.020 (3)
C18	0.061 (3)	0.056 (3)	0.065 (4)	0.007 (2)	0.006 (3)	0.012 (3)
C19	0.075 (4)	0.060 (3)	0.054 (3)	0.015 (3)	0.009 (3)	0.012 (3)
C20	0.063 (4)	0.063 (3)	0.064 (4)	0.015 (3)	0.015 (3)	0.028 (3)
C21	0.050 (3)	0.080 (4)	0.082 (5)	0.005 (3)	0.005 (3)	0.025 (3)
C22	0.061 (3)	0.071 (3)	0.057 (3)	0.011 (3)	−0.002 (3)	0.022 (3)
C23	0.071 (4)	0.098 (4)	0.104 (5)	0.014 (3)	0.031 (4)	0.029 (4)
C24	0.068 (4)	0.046 (3)	0.059 (4)	0.000 (2)	0.003 (3)	0.018 (3)
C25	0.053 (3)	0.052 (3)	0.057 (3)	0.006 (2)	0.006 (3)	0.024 (3)
C26	0.084 (4)	0.047 (3)	0.070 (4)	0.015 (3)	0.026 (4)	0.018 (3)
C27	0.079 (4)	0.040 (3)	0.072 (4)	0.008 (3)	0.021 (3)	0.023 (3)
C28	0.055 (3)	0.043 (3)	0.057 (4)	0.007 (2)	0.006 (3)	0.022 (3)
C29	0.065 (3)	0.050 (3)	0.063 (4)	0.013 (2)	0.006 (3)	0.019 (3)
C30	0.063 (3)	0.047 (3)	0.066 (4)	0.004 (2)	0.009 (3)	0.018 (3)
C31	0.062 (3)	0.060 (3)	0.070 (4)	0.019 (3)	0.009 (3)	0.031 (3)
C32	0.078 (4)	0.090 (4)	0.104 (5)	0.034 (3)	0.034 (4)	0.058 (4)

### Geometric parameters (Å, °)

**Table:**

O1—C15	1.223 (6)	C11—H11	0.9300
O2—C31	1.237 (6)	C13—H13	0.9300
N1—C1	1.423 (6)	C14—H14	0.9300
N1—C8	1.265 (7)	C16—H16C	0.9600
N2—C12	1.415 (6)	C16—H16A	0.9600
N2—C15	1.355 (6)	C16—H16B	0.9600
N2—H2A	0.8600	C17—C22	1.378 (7)
N3—C24	1.255 (6)	C17—C18	1.373 (7)
N3—C17	1.425 (6)	C18—C19	1.398 (7)
N4—C28	1.399 (6)	C19—C20	1.387 (8)
N4—C31	1.349 (6)	C20—C21	1.362 (9)
N4—H4	0.8600	C20—C23	1.521 (9)
C1—C2	1.382 (7)	C21—C22	1.378 (8)
C1—C6	1.384 (7)	C24—C25	1.454 (7)
C2—C3	1.383 (7)	C25—C30	1.376 (7)
C3—C4	1.373 (7)	C25—C26	1.398 (7)
C4—C5	1.373 (7)	C26—C27	1.382 (8)
C4—C7	1.518 (8)	C27—C28	1.406 (7)
C5—C6	1.383 (7)	C28—C29	1.384 (7)
C8—C9	1.467 (7)	C29—C30	1.381 (7)
C9—C10	1.385 (7)	C31—C32	1.501 (8)
C9—C14	1.379 (7)	C18—H18	0.9300
C10—C11	1.375 (7)	C19—H19	0.9300
C11—C12	1.379 (7)	C21—H21	0.9300
C12—C13	1.391 (7)	C22—H22	0.9300
C13—C14	1.397 (7)	C23—H23A	0.9600
C15—C16	1.514 (6)	C23—H23B	0.9600
C2—H2	0.9300	C23—H23C	0.9600
C3—H3	0.9300	C24—H24	0.9300
C5—H5	0.9300	C26—H26	0.9300
C6—H6	0.9300	C27—H27	0.9300
C7—H7C	0.9600	C29—H29	0.9300
C7—H7A	0.9600	C30—H30	0.9300
C7—H7B	0.9600	C32—H32A	0.9600
C8—H8	0.9300	C32—H32B	0.9600
C10—H10	0.9300	C32—H32C	0.9600
			
C1—N1—C8	119.8 (4)	H16A—C16—H16B	109.00
C12—N2—C15	128.0 (4)	C15—C16—H16A	109.00
C15—N2—H2A	116.00	C15—C16—H16C	109.00
C12—N2—H2A	116.00	C15—C16—H16B	110.00
C17—N3—C24	120.6 (4)	N3—C17—C22	124.5 (4)
C28—N4—C31	127.5 (3)	N3—C17—C18	117.0 (4)
C31—N4—H4	116.00	C18—C17—C22	118.4 (5)
C28—N4—H4	116.00	C17—C18—C19	120.9 (5)
N1—C1—C6	123.5 (5)	C18—C19—C20	120.2 (5)
N1—C1—C2	117.9 (4)	C19—C20—C21	117.8 (5)
C2—C1—C6	118.6 (5)	C19—C20—C23	120.6 (5)
C1—C2—C3	119.8 (5)	C21—C20—C23	121.6 (5)
C2—C3—C4	122.4 (5)	C20—C21—C22	122.3 (5)
C3—C4—C7	121.9 (5)	C17—C22—C21	120.2 (5)
C5—C4—C7	121.0 (5)	N3—C24—C25	123.2 (4)
C3—C4—C5	117.1 (5)	C24—C25—C30	121.1 (5)
C4—C5—C6	122.1 (5)	C26—C25—C30	118.3 (5)
C1—C6—C5	120.0 (5)	C24—C25—C26	120.6 (5)
N1—C8—C9	122.2 (5)	C25—C26—C27	120.3 (5)
C10—C9—C14	118.6 (5)	C26—C27—C28	120.6 (5)
C8—C9—C10	121.2 (4)	N4—C28—C27	117.1 (4)
C8—C9—C14	120.1 (5)	C27—C28—C29	118.9 (5)
C9—C10—C11	120.2 (5)	N4—C28—C29	124.0 (4)
C10—C11—C12	121.4 (5)	C28—C29—C30	119.7 (5)
N2—C12—C11	117.2 (4)	C25—C30—C29	122.3 (5)
C11—C12—C13	119.4 (4)	N4—C31—C32	115.5 (4)
N2—C12—C13	123.3 (4)	O2—C31—N4	121.9 (4)
C12—C13—C14	118.6 (4)	O2—C31—C32	122.7 (5)
C9—C14—C13	121.8 (5)	C17—C18—H18	120.00
N2—C15—C16	114.8 (4)	C19—C18—H18	120.00
O1—C15—C16	121.9 (4)	C18—C19—H19	120.00
O1—C15—N2	123.4 (4)	C20—C19—H19	120.00
C1—C2—H2	120.00	C20—C21—H21	119.00
C3—C2—H2	120.00	C22—C21—H21	119.00
C2—C3—H3	119.00	C17—C22—H22	120.00
C4—C3—H3	119.00	C21—C22—H22	120.00
C6—C5—H5	119.00	C20—C23—H23A	109.00
C4—C5—H5	119.00	C20—C23—H23B	109.00
C1—C6—H6	120.00	C20—C23—H23C	109.00
C5—C6—H6	120.00	H23A—C23—H23B	109.00
H7A—C7—H7C	109.00	H23A—C23—H23C	109.00
H7B—C7—H7C	110.00	H23B—C23—H23C	109.00
C4—C7—H7A	109.00	N3—C24—H24	118.00
C4—C7—H7B	109.00	C25—C24—H24	118.00
C4—C7—H7C	109.00	C25—C26—H26	120.00
H7A—C7—H7B	109.00	C27—C26—H26	120.00
N1—C8—H8	119.00	C26—C27—H27	120.00
C9—C8—H8	119.00	C28—C27—H27	120.00
C11—C10—H10	120.00	C28—C29—H29	120.00
C9—C10—H10	120.00	C30—C29—H29	120.00
C10—C11—H11	119.00	C25—C30—H30	119.00
C12—C11—H11	119.00	C29—C30—H30	119.00
C14—C13—H13	121.00	C31—C32—H32A	109.00
C12—C13—H13	121.00	C31—C32—H32B	109.00
C9—C14—H14	119.00	C31—C32—H32C	109.00
C13—C14—H14	119.00	H32A—C32—H32B	109.00
H16A—C16—H16C	109.00	H32A—C32—H32C	109.00
H16B—C16—H16C	109.00	H32B—C32—H32C	109.00
			
C8—N1—C1—C2	141.7 (5)	C10—C9—C14—C13	1.0 (7)
C8—N1—C1—C6	−37.7 (7)	C9—C10—C11—C12	−0.4 (7)
C1—N1—C8—C9	178.8 (4)	C10—C11—C12—N2	−174.8 (4)
C15—N2—C12—C11	−160.9 (4)	C10—C11—C12—C13	1.1 (7)
C15—N2—C12—C13	23.5 (7)	N2—C12—C13—C14	174.9 (4)
C12—N2—C15—O1	5.3 (7)	C11—C12—C13—C14	−0.7 (7)
C12—N2—C15—C16	−174.5 (4)	C12—C13—C14—C9	−0.4 (7)
C24—N3—C17—C18	−146.3 (5)	N3—C17—C18—C19	178.5 (4)
C24—N3—C17—C22	35.9 (7)	C22—C17—C18—C19	−3.6 (8)
C17—N3—C24—C25	179.1 (4)	N3—C17—C22—C21	179.7 (5)
C31—N4—C28—C27	−154.6 (5)	C18—C17—C22—C21	1.9 (8)
C31—N4—C28—C29	25.5 (7)	C17—C18—C19—C20	1.5 (8)
C28—N4—C31—O2	−0.4 (7)	C18—C19—C20—C21	2.2 (8)
C28—N4—C31—C32	178.7 (4)	C18—C19—C20—C23	−178.1 (5)
N1—C1—C2—C3	180.0 (5)	C19—C20—C21—C22	−4.0 (9)
C6—C1—C2—C3	−0.6 (8)	C23—C20—C21—C22	176.4 (6)
N1—C1—C6—C5	−178.9 (5)	C20—C21—C22—C17	1.9 (9)
C2—C1—C6—C5	1.7 (8)	N3—C24—C25—C26	8.0 (8)
C1—C2—C3—C4	0.2 (8)	N3—C24—C25—C30	−169.8 (5)
C2—C3—C4—C5	−0.9 (8)	C24—C25—C26—C27	−178.0 (5)
C2—C3—C4—C7	−179.5 (5)	C30—C25—C26—C27	0.0 (8)
C3—C4—C5—C6	2.0 (8)	C24—C25—C30—C29	176.9 (5)
C7—C4—C5—C6	−179.3 (5)	C26—C25—C30—C29	−1.0 (8)
C4—C5—C6—C1	−2.5 (8)	C25—C26—C27—C28	1.1 (8)
N1—C8—C9—C10	−8.0 (8)	C26—C27—C28—N4	179.0 (5)
N1—C8—C9—C14	169.9 (5)	C26—C27—C28—C29	−1.1 (8)
C8—C9—C14—C13	−176.9 (5)	N4—C28—C29—C30	180.0 (4)
C8—C9—C10—C11	177.3 (5)	C27—C28—C29—C30	0.1 (8)
C14—C9—C10—C11	−0.7 (7)	C28—C29—C30—C25	0.9 (8)

### Hydrogen-bond geometry (Å, °)

**Table:**

*D*—H···*A*	*D*—H	H···*A*	*D*···*A*	*D*—H···*A*
N2—H2A···O2^i^	0.86	2.00	2.854 (5)	172
N4—H4···O1^ii^	0.86	2.06	2.911 (4)	173
C13—H13···O1	0.93	2.40	2.922 (6)	116
C29—H29···O2	0.93	2.35	2.864 (6)	114

Symmetry codes: (i) *x*+2, *y*, *z*−1; (ii) *x*−2, *y*−1, *z*+1.
